# Notes and News

**Published:** 1901-03-09

**Authors:** 


### NOTES AND NEWS.

HOSPITAL MEETINGS.

Royal Westminster Ophthalmic Hospital.—Annual meeting, Friday, March 8th, 4 p.m.

French Hospital.—Annual meeting, Thursday, March 14th, 6 p.m.

THE King has shown his genuine interest in institutions for the care of the sick by visiting two sanatoria for consumption and one hospital during his short visit to Germany. He has already shown his practical sympathy with the movement to introduce the open-air treatment for consumption into England, and his second visit to Falkenstein proves that his interest is unabated.

A MOST unfortunate mistake was recently made by a doctor in Swansea, whereby a man lost his life. The doctor gave the patient some carbolic acid in mistake for medicine, one draught of which caused the fatality.

THE Cadets of the *Britannia* training ship at Dartmouth are enjoying three weeks holiday whilst the ship is being thoroughly disinfected. An epidemic of influenza has been present in the ship which has resulted in two deaths.

THE Countess of Londesborough has just opened a Cottage Hospital at Selby. It has been erected through the generosity of Mr. and Mrs. Goldsborough who gave £1,200, and a further sum of £1,000 was contributed by the Marriott Trustees. The building is two storeys high; it contains an accident ward twenty-two feet by twenty-six feet, and on the upper floor three bedrooms and a bathroom.

MR. KENNETH W. GOADBY, M.R.C.S., L.R.C.P., L.D.S.R.C.S., and Mr. James Warburton Brown, M.R.C.S., L.R.C.P., L.D.S.R.C.S., have been appointed respectively Senior and Junior Dental Surgeon to the Seamen's Hospital Society. Mr. Goadby will take duty at the Branch Hospital of this Society in the Royal Victoria and Albert Docks (to which is attached the London School of Tropical Medicine) and Mr. Warburton Brown will attend at the "Dreadnought" Hospital, Greenwich.

THE diphtheria wing at the Bletchingly Hospital has been burnt down. The patients were safely removed, although the fire spread with great rapidity.

THE people of Glasgow are becoming alarmed lest the epidemic of small-pox which is now nearly over should affect attendance at the Exhibition to open in May. Alarming reports have reached the Continent, which it is feared may affect foreign visitors. Full publicity is to be given respecting all cases, and vaccination is to receive the utmost encouragement.

THE announcement of Dr. V. Jarre to the French Academy of Medicine that he has discovered a remedy for foot-and-mouth disease will be received with interest. The remedy consists of a solution of chromic acid employed as a caustic. Dr. Jarre stated that his experiments had covered a period of two years. The French Minister of Agriculture has promised to give the reported remedy an official test.

THE enormous cost of erecting buildings for the care of the afflicted is illustrated in the case of the proposed lunatic asylum for Edinburgh. The estimates amount to about £380 per bed or upwards of £280,000 for the 1000 beds. This is exclusive of site, costing about £21,000, of a railway for the conveyance of materials for building, &c. at a cost of £33,000, and of a water supply estimated at £16,000. The site purchased is at Bangour.

THERE can be no doubt that the excellent system of visiting, followed by a practical report, adopted by The Prince of Wales's Hospital Fund in the case of all hospitals it proposes to assist, is of the greatest benefit in drawing attention to the real needs of these institutions. In the case of the Chelsea Hospital for Women, the Fund recommended new flooring in the wards. In consequence of this the Mercers' Company has given £26 5s., and the Leathersellers' Company £10 10s. towards this object.

GREATER confidence is felt at the Cape now that the Government has taken into its own hand the suppression of the plague epidemic. Contacts and suspects are now placed in strict quarantine. Dr. Simpson, who has had great experience of the disease in India, has been placed in charge of the sanitary arrangements. The troops are now landed at Simonstown, and passengers to England are medically examined previous to leaving the Cape. One of the vessels proceeding to Sydney from the Cape is reported to have landed a seaman suffering from the disease. The ship was placed in quarantine. A case of plague was also reported at Brisbane.

THE annual public meeting of the After Care Association for Poor Persons discharged Recovered from Asylums for the Insane, which had been fixed for January 29th at Lambeth Palace, having had to be postponed owing to the death of Her late Majesty Queen Victoria, the Council decided not to hold a public meeting this year but only a general business meeting; this took place at the Church House, Dean's Yard, Westminster, on February 21st, Dr. Henry Rayner (Chairman of the Council) in the chair. The report showed that good and satisfactory work had been done during the past year but funds are much required to extend and develop the work.

